# Multiscale analysis of lignocellulose recalcitrance towards OrganoCat pretreatment and fractionation

**DOI:** 10.1186/s13068-020-01796-8

**Published:** 2020-09-05

**Authors:** Dennis Weidener, Murali Dama, Sabine K. Dietrich, Benedict Ohrem, Markus Pauly, Walter Leitner, Pablo Domínguez de María, Philipp M. Grande, Holger Klose

**Affiliations:** 1 Institute of Bio- and Geosciences, IBG-2: Plant Sciences, Forschungszentrum Jülich, Leo- Brandt-Straße, 52425 Jülich, Germany; 2 Institute of Technical and Macromolecular Chemistry (ITMC), RWTH Aachen University, Worringer Weg 1, 52074 Aachen, Germany; 3 Bioeconomy Science Center (BioSC) C/O Forschungszentrum Jülich, 52425 Jülich, Germany; 4 Institute for Plant Cell Biology and Biotechnology, Heinrich Heine University, Universitätsstraße. 1, 40225 Düsseldorf, Germany; 5 Institute of Biology I, RWTH Aachen University, Worringerweg 1, 52074 Aachen, Germany; 6 Max Planck Institute for Chemical Energy Conversion, Stiftstraße 34-36, 45470 Mülheim an Der Ruhr, Germany; 7 Sustainable Momentum, SL. Av. Ansite 3, 4-6. 35006, Las Palmas De Gran Canaria, Spain

**Keywords:** Lignocellulose recalcitrance, Biomass, Cell wall analysis, Pretreatment, OrganoCat, 2D-HSQC-NMR

## Abstract

**Background:**

Biomass recalcitrance towards pretreatment and further processing can be related to the compositional and structural features of the biomass. However, the exact role and relative importance to those structural attributes has still to be further evaluated. Herein, ten different types of biomass currently considered to be important raw materials for biorefineries were chosen to be processed by the recently developed, acid-catalyzed OrganoCat pretreatment to produce cellulose-enriched pulp, sugars, and lignin with different amounts and qualities. Using wet chemistry analysis and NMR spectroscopy, the generic factors of lignocellulose recalcitrance towards OrganoCat were determined.

**Results:**

The different materials were processed applying different conditions (e.g., type of acid catalyst and temperature), and fractions with different qualities were obtained. Raw materials and products were characterized in terms of their compositional and structural features. For the first time, generic correlation coefficients were calculated between the measured chemical and structural features and the different OrganoCat product yields and qualities. Especially lignin-related factors displayed a detrimental role for enzymatic pulp hydrolysis, as well as sugar and lignin yield exhibiting inverse correlation coefficients. Hemicellulose appeared to have less impact, not being as detrimental as lignin factors, but xylan-*O*-acetylation was inversely correlated with product yield and qualities.

**Conclusion:**

These results illustrate the role of generic features of lignocellulosic recalcitrance towards acidic pretreatments and fractionation, exemplified in the OrganoCat strategy. Discriminating between types of lignocellulosic biomass and highlighting important compositional variables, the improved understanding of how these parameters affect OrganoCat products will ameliorate bioeconomic concepts from agricultural production to chemical products. Herein, a methodological approach is proposed.

## Background

Lignocellulosic biomass (or the plant cell wall) is the most abundant renewable resource on earth. It is mainly composed of polysaccharides—such as cellulose and various hemicelluloses—and the aromatic macromolecule lignin. To replace fossil resources, lignocellulose has the potential to be converted to alternative fuels, chemicals, or materials without compromising global food security [1]. Generally, lignocellulose structure can be described as cellulose fibrils embedded in a matrix of non-cellulosic polysaccharides and lignin providing strength and mechanical support to the plant [2]. This complex structural and compositional nature of the material often hampers an efficient fractionation, a phenomenon known as recalcitrance. Lignocellulose recalcitrance depends on the heterogeneous multiscale structure of the wall materials and impacts the subsequent conversion process [3, 4]. Despite many studies on lignocellulose recalcitrance, it is still unclear how the structural and chemical features hamper a successful and efficient biomass pretreatment [5]. Many factors have been related to the recalcitrance of the plant cell walls: Changes in the cellulose´s degree of polymerization, its crystallinity, and pore size can interfere with the hydrogen-bond network of the fibers and therefore are believed to facilitate accessibility of degrading enzymes [6]. Hemicelluloses—mainly xylan—can have chemical modifications such as substituents. Hemicelluloses are amorphous and are relatively well hydrolyzed by, e.g., dilute acids in pretreatment processes [5]. These hemicelluloses represent a physical barrier limiting accessibility of the cellulosic fiber. Their removal during the pretreatment can enhance the conversion of cellulose by improving the accessibility of cellulose to enzymes [7, 8]. Moreover, in nature, hemicellulose occurs esterified with acetyl groups [9, 10]. *O*–acetylation might sterically hamper the productive binding of cellulases. For instance, a reduced acetyl content in corn stover seems to improve effectiveness of these enzymes [11], but might still depend strongly on the chemical structure of xylan and/or the interaction with other wall polymers [5].

Finally, lignin is a complex heterogeneous polyphenolic macromolecule and has been described in many studies to severely enhance recalcitrance of lignocellulose deconstruction. It is responsible for hydrophobicity, structural rigidity, and brittleness of wall materials, and it is connected through the hemicelluloses to cellulose inside the walls [12, 13]. Lignin consists of phenylpropanoid building units: *p*-coumaryl (H), coniferyl (G), and sinapyl alcohol (S). The S and G units form the backbone of the polymer via *β*-aryl ether (β-*O*-4) bonds. In addition, carbon–carbon bonds such as β–β, 5–5, and β–5 linkages are also formed between these lignin units [14]. Lignin content, structure, subunits, and linkages with polysaccharides can vary greatly among different plant species and tissues. These lignin structural parameters are of considerable importance for lignocellulose recalcitrance [15].

The lignin S/G ratio has shown particular influence on pretreatment and the enzymatic hydrolyzability of pretreated biomass [15]. Different studies reported an enhanced saccharification based on S/G ratios, e.g., on poplar by thermochemical pretreatments [16, 17]. A preferential removal of S relative to G-lignin units in poplar was observed during flow through pretreatment [18]. In addition, the S/G ratio is positively related with delignification by green liquor and Kraft pulping of poplar [19, 20]. The main reasons influencing lignin depolymerization are that S-lignin exhibits a higher level of β-*O*-4 linkages, which are more prone to cleavage during the pretreatment and delignification caused by the lower molecular weight of S-lignin and lower crosslinking, simplifying lignin removal [15]. However, this dependence does not seem to be universally valid, since no clear correlation was observed using Kraft pretreatment on eucalyptus [21, 22]. This interdependence does not become clearer when studying the remaining lignin residue in pretreated biomass. S-lignin with its higher number of β-*O*-4 linkages tends to form a linear configuration and, therefore, covers more of the cellulose fibers [23]. In contrast, the higher density of linkages in G-lignin provides a stronger physical barrier [24]. These partly contradictory results suggest a much more complicated relationship between S/G ratio and lignocellulose recalcitrance. The multidimensional nature of plant´s species and pretreatment with the accompanying changes of the entire cell wall structure most likely masks the effects resulting from the lignin composition in many cases [15].

Many prominent examples demonstrate the effective, economically competitive utilization concepts for lignocellulose in biorefineries [25]. A full valorization of lignocellulose will be a decisive economic and ecological advantage. The separation and valorization of all three polymer groups can target a broad range of applications such as bio-derived fuels, chemicals, and materials. In recent years, particular progress on lignin valorization has been made while simultaneously utilizing the polysaccharides [26, 27]. While an efficient fractionation and subsequent component valorization is desirable, the ability to successfully recycle the water, solvents, and catalysts used in the pretreatment is crucial for the economic viability, as well. To circumvent these issues, the OrganoCat technology utilizes an organic acid as catalyst at 125–160 °C to mildly hydrolyze the amorphous non-cellulosic sugar polymers, minimizing monosaccharide degradation such as dehydration and subsequent oligomerization. Lignin is extracted in situ into a second phase, 2-methyltetrahydrofuran (2-MTHF), removing it from the reactive phase to diminish condensation reactions. The pulp, now enriched in cellulose, remains as a solid in the aqueous phase and can be filtered off and thus recovered. The three resulting product streams, cellulose-enriched pulp, hydrolyzed monosaccharides, and lignin can be separated and the water, solvent, and catalyst recycled [28–30].

In this work, we investigated the relationship between the plant wall composition of ten different lignocellulosic materials and its role in an acidic pretreatment, using OrganoCat as prototypical approach for full valorization. Aspects such as enzymatic degradation of the pulp after pretreatment, hemicellulose hydrolysis, and lignin yield and quality are assessed. A comprehensive compositional and structural characterization of polysaccharide and lignin structural features is performed to provide an in-depth understanding of the roles of the structural features of lignocellulose in recalcitrance towards pretreatment and fractionation.

## Results and discussion

Ten different types of biomass from diverse sources were chosen representing potentially important raw materials for biorefineries: Four different perennial plants, Miscanthus, Sida, Silphium, and Szarvasi, industrial crop residues, Rapeseed straw, Sugarcane bagasse, Corn cobs (Maize cobs) and Pineapple (without fruit), and two woody biomasses, Beech wood and Eucalyptus, were investigated. In addition, we included data from our previous study on EFB in the multivariate data analysis [31]. The materials were characterized based on their performance in OrganoCat processing. Unprocessed and pretreated samples were analyzed using NMR spectroscopy and various chemical methods to gain further insight into lignocellulosic structure and its recalcitrance towards OrganoCat pretreatment.

### Pretreatment and fractionation of different types of biomass

OrganoCat pretreatment was conducted using two different catalysts: (1) oxalic acid (140 °C, 3 h, 0.1 M oxalic acid referred to water volume) and (2) 2,5-furandicarboxylic acid (3 h, 0.1 M FDCA in both cases referred to water volume) at three different temperatures (140 °C, 150 °C, and 160 °C) due to FDCA’s higher thermal stability compared to oxalic acid [30]. For each set of conditions, triplicates were produced to assure robustness and reproducibility of the results. Depending on the lignocelluloses, significant differences in fractionation efficiency—lignin separation, hemicellulose hydrolysis, and pulp accessibility—were observed when comparing the process for the different materials (Table 1). For all materials, expectedly, the amount of remaining pulp decreases when applying higher process temperatures as more sugars and lignin are extracted from the biomass. Nevertheless, with an increased extraction, the accessibility of the cellulose-enriched pulp for enzymatic hydrolysis increased, as well.

**Table 1:** Pretreatment and fractionation of different lignocellulosic materials

	Highest pulp yield [wt %]^a^	Highest pulp hydrolysis [Glc yield wt %]^b^	Highest sugar yield [wt %]^a^	Highest lignin yield [wt %]^a^	Temperature dependence [-]^c^
Pineapple	51.7 ± 1.6	53.2 ± 2.2	26.4 ± 1.9	16.5 ± 2.5	− 0.36
Beech wood	64.5 ± 0.7	28.0 ± 2.0	26.3 ± 2.4	16.7 ± 2.1	− 0.78
Eucalyptus	62.2 ± 3.7	32.6 ± 3.3	15.3 ± 1.1	0.4 ± 0.1	− 0.57
Maize cobs	39.5 ± 0.6	56.0 ± 2.6	30.1 ± 0.5	0.6 ± 0.1	− 0.46
Miscanthus	70.2 ± 0.2	38.6 ± 6.0	17.9 ± 0.9	15.3 ± 0.9	− 0.78
Rapeseed	54.9 ± 2.5	55.4 ± 2.2	10.5 ± 1.9	10.0 ± 2.3	− 0.37
Sida	67.3 ± 1.0	44.6 ± 3.8	16.4 ± 2.0	13.2 ± 3.3	− 0.71
Silphium	54.0 ± 2.2	37.4 ± 0.3	5.9 ± 0.7	15.0 ± 2.3	− 0.75
Sugarcane bagasse	56.3 ± 1.3	60.8 ± 4.1	17.3 ± 2.6	18.7 ± 2.3	− 0.55
Szarvasi	42 ± 0.9	65.1 ± 1.3	24.8 ± 1.6	19.4 ± 1.2	− 0.52

Depicting the highest yields obtained for the three OrganoCat products over all conditions and for ten different lignocellulosic materials (for full data set, see Additional file 1: Table S1). Yields are expressed in percentage of initial dried material, as mean ± standard deviation; *n* = 3

^a^Percentage by weight in relation to the initial biomass

^b^Percentage by weight in relation to pulp

^c^Temperature dependence is defined as slope of the linear regression between the different glucose release values from enzymatic hydrolysis of pulps after 72 h for FDCA pretreatment at 140, 150, and 160 °C

**Fig. 1:**
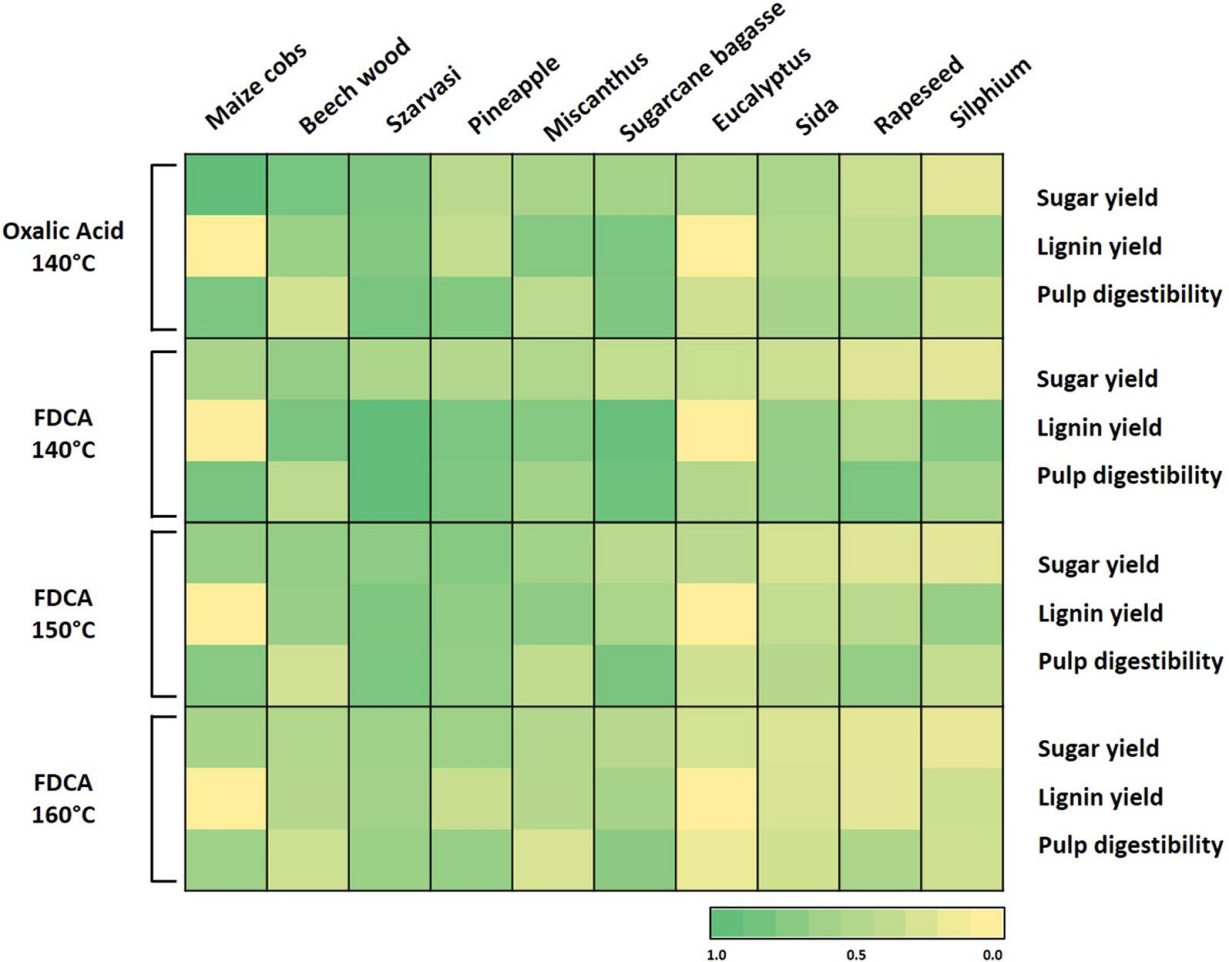
Heat map of the OrganoCat pretreatment and fractionation results for the ten different types of lignocellulosic plant materials under the different process conditions. Color density represents normalized values for the amount of sugars and lignin yield and pulp digestibility. Sugar yield is defined as the amount of reducing sugars determined in the aqueous phase after pretreatment. Lignin yield is defined as the amount of lignin extracted with 2-MTHF after pretreatment. Pulp digestibility is defined as glucose release from cellulase digestion of the pulp after pretreatment (for full data set, see Additional file 1: Table S1)

In general, we could observe that at the highest temperature (160 °C), a similar amount of hydrolyzed sugars was measured compared to 150 °C (Additional file 1: Table S1). Due to the higher temperature, a larger quantity of the sugars is presumably converted to furans and, therefore, the observed amount of sugars did not increase, which is consistent with the previous research [30]. OrganoCat processing aims at full valorization of all three product streams. Comparing the three products, processed from the lignocellulosic materials at the different conditions, we observed that the materials exhibited differences in susceptibility to the pretreatment. Especially, Szarvasi biomass and Sugarcane bagasse seemed to be well suited for the complete valorization, since it exhibited high values for all three product parameters in agreement with our previous work [32]. In contrast, Eucalyptus was very recalcitrant towards OrganoCat with only low amounts of extracted lignin and sugars and minor changes in enzyme digestibility of the pulp (Fig. 1). To identify molecular parameters connected with the processability by OrganoCat, we performed a detailed analysis in terms of the composition and structure of all raw materials and products (Additional file 1: Tables S2, S3).

### Compositional analysis of raw materials and OrganoCat products

**Fig. 2:**
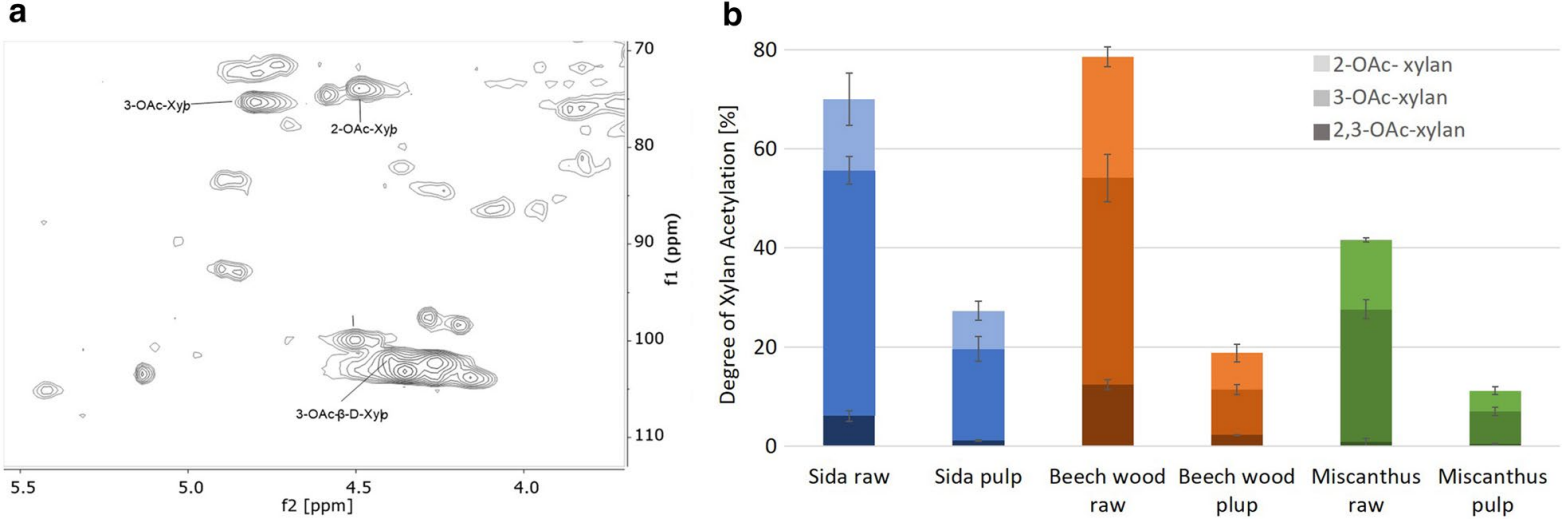
Xylan deacetylation during OrganoCat pretreatment. **a** ^1^H-^13^C HSQC-NMR of untreated Miscanthus biomass indicated the signals used to quantify the degree of xylan acetylation of the biomass. **b** Comparison of xylan-*O*-acetylation of Sida, Beech wood, and Miscanthus before and after pretreatment. 2-*O*Ac is indicated by light color, 3-*O*Ac by medium color and 2,3-*O*Ac by dark color. Values are expressed as mean ± standard deviation; *n* = 3

To illustrate the observed changes in lignocellulose composition produced by the OrganoCat pretreatment, an exemplary subset of Miscanthus, Beech wood, and Sida biomass was subsequently evaluated in detail on the compositional changes of the biomass during the pretreatment with oxalic acid at 140 °C for 3 h. These lignocellulosic materials are considered as prototypic for different phylogenetic groups in this study. The compositional analysis revealed the removal of xylose as the major constituent sugar of hemicellulose in the different materials and additionally an increase in the relative cellulose (glucose) content in the different pulps. Thus, the ratio between glucose and xylose comparing pulp and biomass shifted in case of Sida from 1.5 to 3.5, for Beech wood from 1.8 to 3.2 and for Miscanthus from 1.6 to 3.3, respectively. Higher severity might even increase xylan hydrolysis and, therefore, result in a more extensive xylose removal from the pulp, but it also triggers the production of furans from sugars at harsher conditions [33, 34]. Besides the hemicellulose depolymerization by selective hydrolysis of glycosidic linkages, acidic conditions and temperature during the OrganoCat also liberated some *O*-acetyl groups from the lignocellulosic moieties, mainly from xylan acetylation (Fig. 2).

The released acetic acid during the pretreatment co-catalyzes and enhances hemicellulose depolymerization [35]. OrganoCat, like other diluted acid pretreatments, removes some of the *O*-acetyl moieties. In Beech wood, the degree of xylan-O-acetylation is reduced from 78.6% (raw material) to 18.8% (pulp) upon pretreatment. Considering that 63% of xylan is removed during the pretreatment, a substantial amount of acetic acid is released during the OrganoCat process. In all examined reactions, the *O*-acetates in the 3-position of xylan were deacetylated at a higher proportion than the *O*-acetates in the 2-position (Fig. 2b), which is consistent with the previous findings [31, 36]. Acetyltransferases catalyze the addition of *O*-acetyl groups to the 2-position of the xylosyl residues in xylan followed by non-enzymatic acetyl migration to the O-3 position. The acetylation pattern of xylan depends on the developmental stage of the plant and is interchangeable [37].

**Fig. 3:**
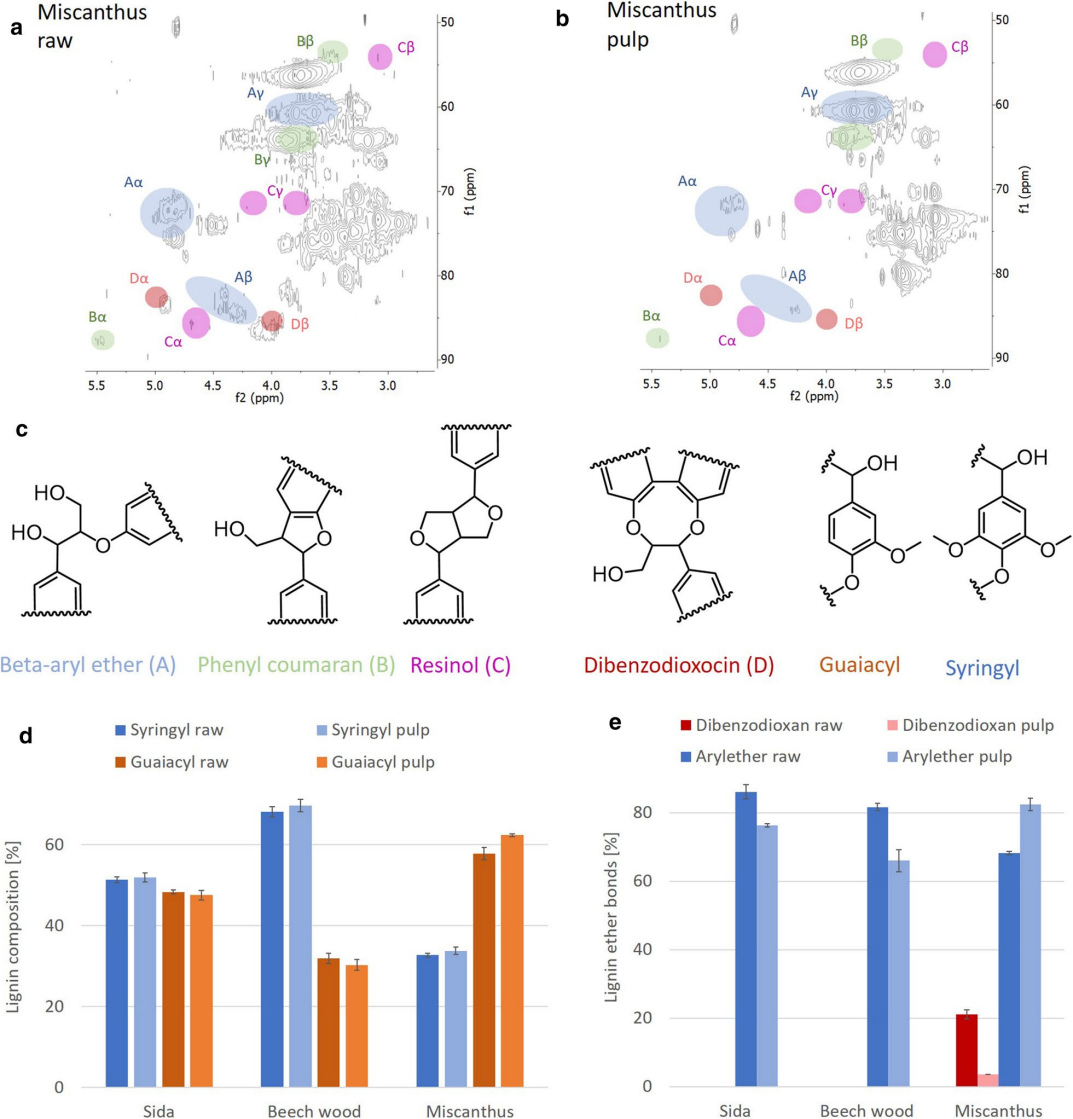
Changes in lignin structure and composition during OrganoCat pretreatment. **a** 2D HSQC spectra of Miscanthus raw material and **b** pulp (oxalic acid, 140 °C highlighting the signals used for quantification. **c** Chemical structure of examined lignin linkages and monomers. **d** Lignin composition showing the Guaiacyl (G) and Syringyl (S) molar distribution for Sida, Beech wood, and Miscanthus. **e** Molar distribution of lignin ether bonds for Sida, Beech wood, and Miscanthus. Values are expressed as mean ± standard deviation; *n* = 3

Lignin was investigated using 2D HSQC-NMR spectroscopy for the identification of unit composition and linkages inside the macromolecule (Fig. 3). In addition, the lignin unit composition was also confirmed using thioacidolysis followed by GC–MS determination. Comparing the unit composition of the monomers before and after pretreatment, no significant changes were observed (Fig. 3d). At higher temperatures, it has been shown that the S/G ratio is reduced due to methoxy groups being eliminated during pretreatment [36, 38]. The proportion of the aryl ether linkages (β-*O*-4) between the monomers decreased for Sida and Beech wood upon pretreatment (Fig. 3e). Ether bonds are more sensitive at increased temperatures and lower pH; therefore, the ether bonds are predominately cleaved, whereas the C–C bonds are hardly affected under these conditions [39].

A noteworthy exception is observed in grass biomass material exemplified by Miscanthus, where an increase in the relative amount of β-*O*-4 linkages after pretreatment could be detected (Fig. 3e). This is most probably due to the fact that grasses exhibit a significant amount of dibenzodioxocin linkages with two ether bonds [40, 41]. If only one of the two ether bonds is cleaved, dibenzodioxocin decreases, but β-*O*-4 increases.

**Fig. 4:**
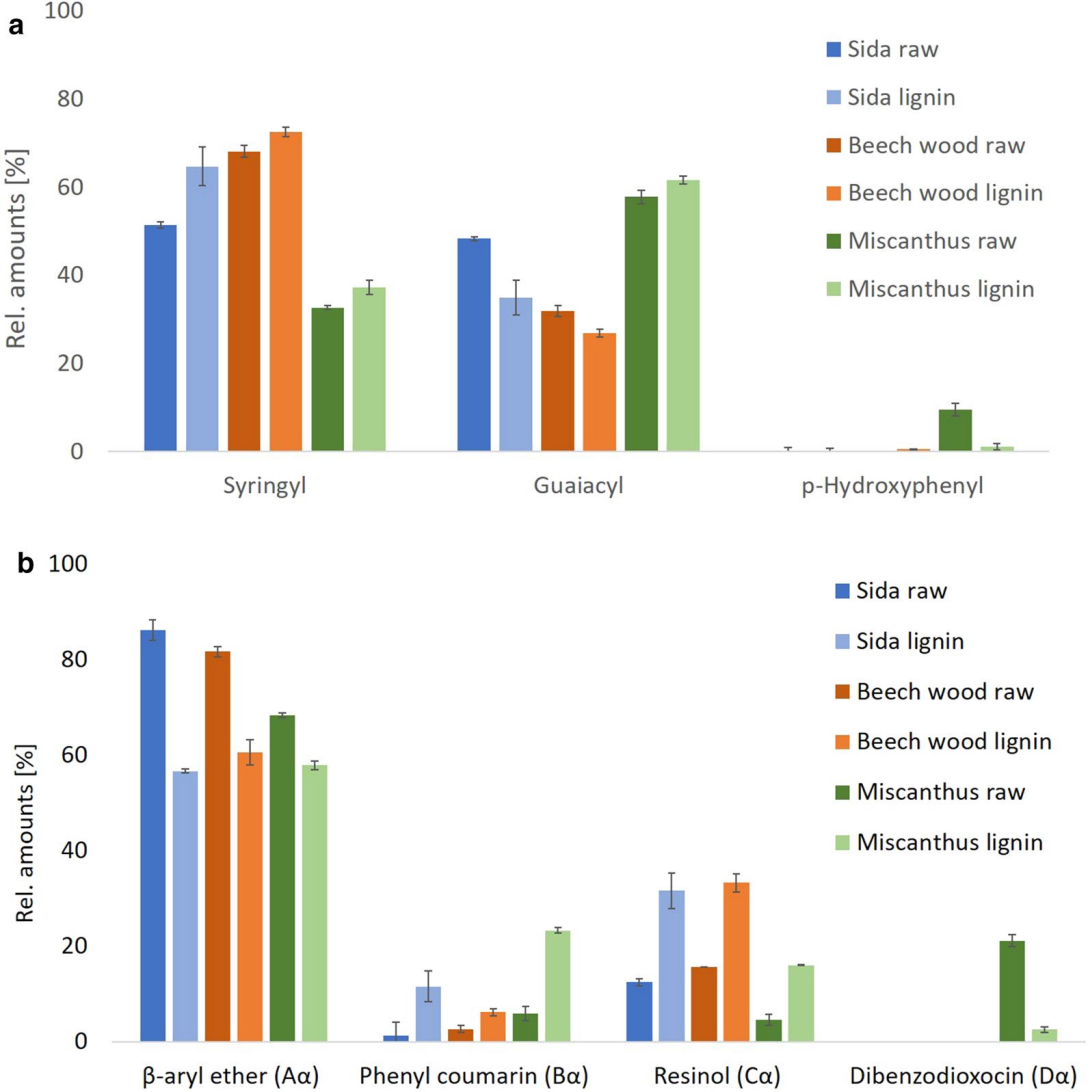
Structural and compositional changes of OrganoCat extracted lignin compared to untreated material. Semi-quantitative analysis by 2D HSQC-NMR to determine the molar ratio between the observed features. **a** Monomer composition in lignocellulosic biomass and in the extracted lignin based on NMR analysis. **b** Lignin linkage distribution in lignocellulosic biomass and in extracted lignin. Values are expressed as mean ± standard deviation; *n* = 3

During the OrganoCat process, lignin is extracted into the 2-MTHF phase, removing it from the reactive phase and, therefore, reducing condensation reactions and maintaining a higher amount of aryl ether linkages [30]. Since these linkages are more prone to subsequent chemical or biochemical reactions, a high content of β-*O*-4 linkages could be beneficial for further conversion and valorization of the extracted lignin [42, 43]. The monomer composition of the extracted lignin did not change in lignin from Beech wood and Miscanthus (Fig. 4a), in line with the previous results for similar processes [7, 30]. Surprisingly, OrganoCat pretreatment conducted for Sida leads to an increase of the S/G ratio in the extracted phase (Fig. 4a). More detailed studies have to be carried out to identify, if differences in the monomer distribution in Sida lignin favor the extraction of lignin enriched in S units by OrganoCat processing.

G units can be additionally interlinked at their C-5 atom via β-5 or 5-5 bond [14, 44]. This might mean that the distribution and linking of the monomers in Sida lignocellulose is less uniform than in the other two plant biomasses. Therefore, the less interlinked, higher S containing lignin is extracted at the mild OrganoCat conditions. Lignin valorization strategies often rely on selective depolymerization of the β-aryl ether bonds. Thus, their retention in the extracted lignin structure is essential [45, 46]. The applied mild pretreatment conditions ensure that a substantial proportion of these C–O bonds is conserved during the process. Nevertheless, we could observe a reduction of the β-*O*-4 motifs in the HSQC spectra for extracted lignin from all plants compared to the raw material.

The resinol motifs increased in the spectra of extracted lignin for the three plants (Fig. 4b). Resinol units are reported to be starting sites for lignin polymerization, and are thus more abundant towards the center of lignin composites. This results in a proportional increase in their abundance as the MW of lignin decreases indicating a tendency to favor extracting smaller lignin fragments [44].

### Multivariate data analysis

**Fig. 5:**
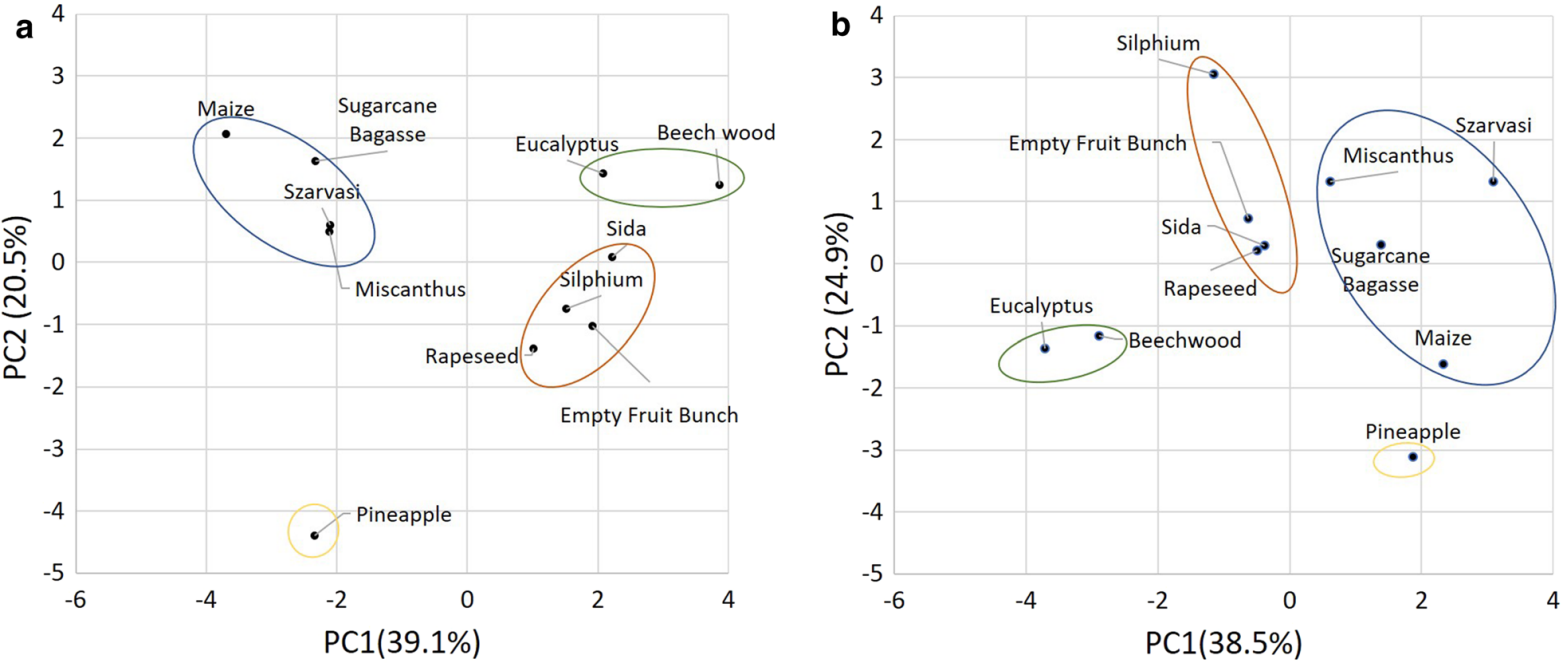
Principle component analysis of raw (**a**) and OrganoCat (**b**) pretreated materials

In addition to the ten characterized plant materials in this study, data from our previous work on pretreatment of empty fruit bunches (*Elaeis guineensis*) [31] were included into the multivariate analysis. A set of 17 normalized parameters (supplementary file) was used to cluster the different biomasses using principle components (Fig. 5). Based on this analysis biomass from the Poaceae family (grasses), Corn (Maize), Sugarcane bagasse, Miscanthus, and Szarvasi were identified as one cluster in this analysis. The second cluster is formed by a phylogenetically more different group, Sida (Malvaceae), Rapeseed (Brassicaceae), Silphium (Asteraceae), and, interestingly, Empty Fruit Bunch material from oil palms, which is phylogenetically closer related to the Poaceae. The two woody materials Beech wood and Eucalyptus form the third cluster and the Pineapple material forms a fourth cluster. A similar clustering was performed using parameters from the pretreatment products (Additional file 1: Tables S4, S5). This analysis indicated a similar clustering as the raw material, although Poaceae group members were further apart. Interestingly, Miscanthus and Szarvasi, which were located closely together in the compositional clustering, are more distant after OrganoCat pretreatment. Comparing the first two principle components by their loadings for both analyses—raw and OrganoCat pretreated materials—the contribution of S-lignin, and S/G ratio for PC1 and G-lignin and β-*O*-4 motifs for PC2 increased significantly outlining the importance of these factors for the OrganoCat pretreatment (Additional file 1: Tables S6, S7).

To some extent, similarities in lignocellulose structure and composition can outline the performance in the whole OrganoCat process. The recalcitrance of the lignocellulosic materials towards the pretreatment and fractionation depends on the interaction of the substrate—catalyst system [3]. Therefore, identifying the key structural features that are responsible for the performance within the OrganoCat would provide valuable information to design an efficient process tailored for different types of input materials. To investigate parameters which exhibit an impact on OrganoCat processing throughout the whole set, coefficients of one-to-one correlations were calculated to highlight lignocellulosic markers with strong impact on pulp saccharification, sugar yield in the aqueous phase, and yield of extracted lignin.

Lignin has an impact on the processability of biomass, for example, being a physical barrier to enzymatic hydrolysis reducing the accessibility of the cellulose towards the enzymes [47]. In addition, lignin binds proteinaceous enzymes with its hydrophobic surfaces removing them from hydrolysis suspension [47]. The molecular composition and structure of lignin plays an important role for biomass recalcitrance and subsequently lignin valorization [26, 43]. In particular, the role of S/G ratio for recalcitrance has been intensively studied [48, 49].

**Fig. 6:**
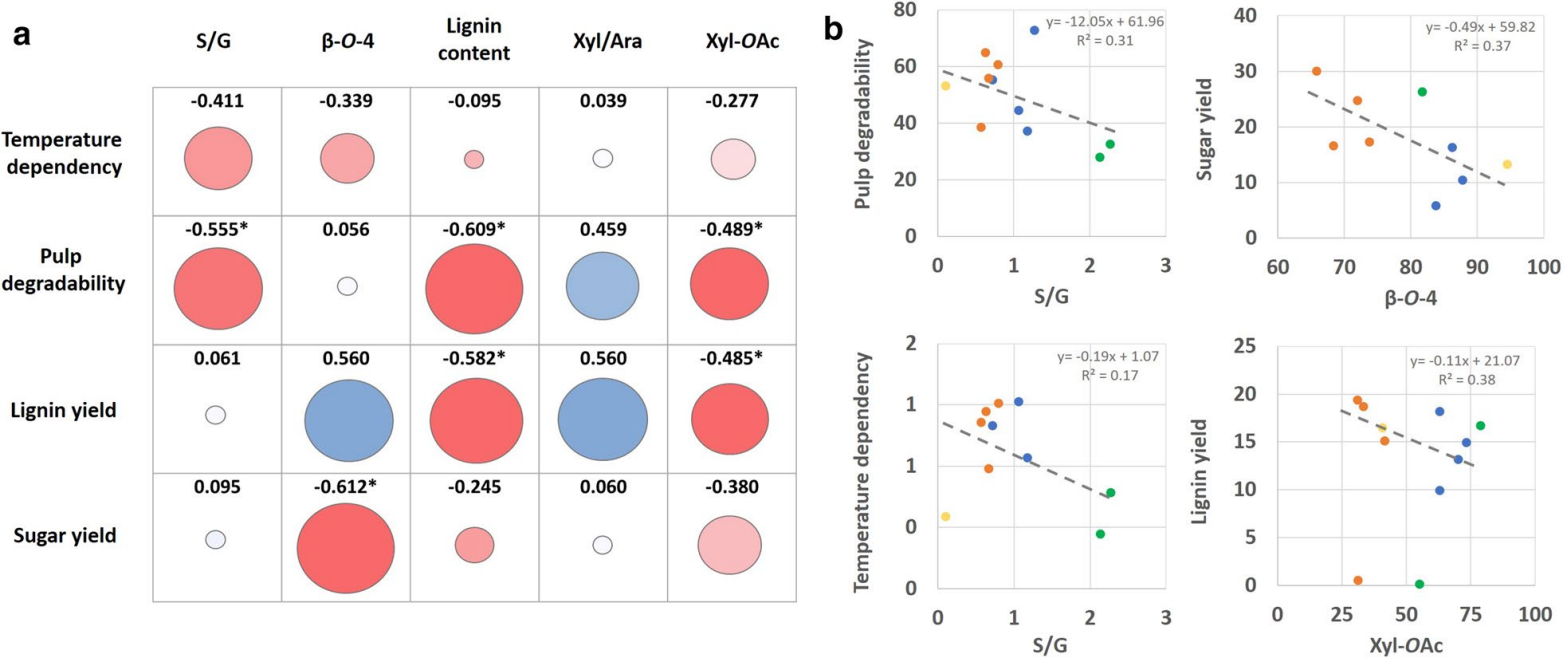
Relationships between measured chemical and structural features and OrganoCat performance parameters. Temperature dependence is defined as the slope of the linear regression between the different glucose release values from enzymatic hydrolysis of pulps after 72 h for FDCA pretreatment at 140, 150, and 160 °C. Pretreatment efficiency is defined as pulp yield after pretreatment using FDCA and 160 °C. Pulp degradability is defined as glucose release from cellulase digestion of the pulp after using FDCA and 160 °C, Sugar yield is defined as the amount of reducing sugars determined in the aqueous phase after pretreatment with FDCA 160 °C. Lignin yield is defined as the amount of lignin extracted with 2-MTHF after pretreatment with FDCA at 160 °C. **a** Pearson’s correlation coefficients between OrganoCat parameters and biomass-related features. Disc diameter and color shade display the strength of the correlations. Positive correlations are displayed in blue and negative red, respectively. Asterisks indicate statistically significant coefficients with a p value ≤ 0.05. **b** Relationship between different lignocellulose parameters and selected parameters for OrganoCat performance. Plant materials are grouped based on the principle component analysis, blue circle: Maize, Sugarcane bagasse, Szarvasi, Miscanthus; orange circle: Sida, Silphium, Rapeseed, Empty fruit bunch; green circle Beech wood, Eucalyptus; yellow circle Pineapple

The dependence on higher temperatures (more severe conditions) to produce pulp which exhibits higher glucose release rates during pulp enzymatic hydrolysis elevates with higher S/G ratio (Fig. 6). In addition, the proportion of β-*O*-4 linkages has a negative correlation with temperature dependence, as well. It has been shown that G units can form a more cross-linked lignin by additional C–C bonds than the S unit, and, thus, can form a greater physical barrier against enzymatic degradation [50]. In our study, we found that a higher S/G was proportional to higher ratio of β-*O*-4 linkages (Additional file 1: Table S8) which is similar to previously published studies [48]. These linkages are more easily cleavable by temperature. Therefore, a lignin with a higher ratio of G units and an elevated level of C–C couplings is less affected by an elevated temperature and would inhibit subsequent enzymatic hydrolysis if it is not separated. For the pulp degradability (FDCA, 160 °C), we could observe a negative correlation with the S/G ratio in untreated biomass. This is supported by our data, showing that the S/G ratio did not change in the pulp compared to the raw material. High lignin content and high S/G ratio result in a high amount of residual pulp. The proportion of β–*O*–4 linkages has only a minor effect in this, which is different for the lignin yield in the organic phase. The positive coefficient indicates the effectiveness of OrganoCat, similar to other organosolv pretreatments, to be partially dependent on the cleavage of β-aryl ether linkages [51, 52]. As expected, biomass with higher content of β–*O*–4 linkages yielded a higher amount of extracted lignin. Davidson et al. reported that for diluted acid pretreatment, a lower S/G ratio in poplar biomass led to a higher yield in xylose [50]. We did not observe this for the sugar yield (manly xylose from the hemicellulose) in our study (Fig. 6a), when using the whole set of lignocellulosic materials. However, when we excluded the extreme low and high S/G ratios (below 0.5 Pineapple, and above 1.5, Beech wood and Eucalyptus), a negative correlation was observed (Pearson coefficient R = − 0.61, p < 0.05) between the S/G ratio and the sugar yield. Since β–*O*–4 linkages and S/G ratio are linked as described above, the inverse correlation of this linkage type with the sugar yield can be explained, respectively. Similar to several studies, which demonstrated a role of the lignin S/G ratio for different diluted acid processes of lignocellulose [48, 50], we can conclude that S/G ratio can serve to some extent as an index to anticipate recalcitrance towards acidic pretreatments like OrganoCat processing.

In addition to lignin-related factors, we could also show that polysaccharide specific parameters correlate with recalcitrance towards OrganoCat. Many cellulose-specific parameters like degree of polymerization and crystallinity index have already been shown to be important for pretreatment and enzymatic hydrolysis of lignocellulose [5]. However, cellulose-specific factors were not part of this investigation. Structural parameters of hemicellulose have also been shown to be important parameter to describe lignocellulose recalcitrance [7, 35, 49]. The combination of xylan with other sugar moieties and acetyl residues are crucial for the structural alignment of the polysaccharide inside the cell wall [53] and, therefore, play a role in the processability of lignocellulose [35, 49]. In our study, the ratio of xylan to arabinose (Xyl/Ara) showed no significant relationship. Wu et al. described a negative correlation for Xyl/Ara for cellulose hydrolysis after acid pretreatment [49], suggesting that the branched Ara of xylans may be interlinked with cellulose chains via hydrogen bonds and, therefore, reduce cellulose crystallinity in the Poaceae Miscanthus, rice, and wheat [54]. If only the four Poaceae plants are considered for the analysis, a negative correlation was observed between cellulose degradability and Xyl/Ara ratio, yet at low significance (Pearson coefficient R = -0.51). The xylan of Poaceae can be linked to lignin by ferulic acid esterified arabinosyl residues [9]. Genetically engineered tall Fescue with lower amounts of these ferulate esters displayed elevated levels of enzymatic digestibility [55]. Therefore, in Poaceae, a high Xyl/Ara ratio (low amount of arabinosyl residues) potentially indicates a reduced number of sites for ester bonds to the lignin and thus reduced recalcitrance.

Interestingly, we observed a negative and significant correlation of the xylan acetylation degree with the pulp degradability and the lignin yield in the organic phase. The deacetylation of corn stover prior to diluted acid pretreatment has been described as favorable [47, 56]. Xylan deacetylation has been described as the rate limiting step for xylose monomer formation during the pretreatment, likely because deacetylated xylans can form aggregates [56], also a crucial step in the OrganoCat process [28].The calculated Pearson coefficient for different pretreatment temperatures (Additional file 1) showed that the correlation diminishes with higher temperatures, underlining the observations.

## Conclusions

The compositional analysis of ten different biomasses processed by OrganoCat pretreatment allowed to determine the correlations between the plant biomasses´ chemical and structural features on one hand and the corresponding OrganoCat product yields and qualities on the other hand. Our results elucidate the role of some generic features of the lignocellulose’s recalcitrance towards this pretreatment and fractionation technology. It was possible to discriminate between the main types of lignocellulosic biomasses such as grass and wood materials and to highlight important variables, especially lignin (S/G) and hemicellulose (xylan-O-acetylation) features. Biomasses from energy grasses or residual straw from crop production appeared to be particularly suitable for OrganoCat processing under the evaluated conditions by being highly predictable towards effective pretreatment and fractionation.

A better understanding of how compositional and structural parameters of lignocellulosic biomass affect the different OrganoCat products subsequently will lead to improved and tailored strategies from agricultural production to chemical products.

## Methods

### Plant biomass material

Biomass from the perennial plants *Sida* *hermaphrodita*, *Silphium* *perfoliatum*, *Elymus* *elongatus* – Szarvasi-1, and *Miscanthus *× *giganteus* originated from a previously described field trail [32]. Maize material (Corn cobs) was obtained from a field in close proximity. Rapeseed straw (Cultivar R53) was produced in the Greenhouse (Institute for Botany, RWTH Aachen University). Pineapple wood was produced in Costa Rica by Agricola Industrial La Lydia. Sugarcane bagasse and Eucalyptus wood were kindly provided by EMBRAPA-Brazil. Beech wood was received from Rettenmaier & Söhne in the form of wood chips (Räuchergold^®^). Sida, Silphium, Szarvasi, Miscanthus Rapeseed, and Maize material was dried for 7 days at 85 °C. Pineapple, sugarcane, and beech wood were obtained already as dried material. All samples were grinded to a fine powder using a ball mill M 400 (Retsch, Haan, Germany) in a 50 mL metal beaker (30 s^−1^, 2 min). For the pretreatment and all analytics, three independent technical replicates were taken from this material.

### Lignocellulose compositional analyses

Wet chemistry-based analysis of lignocellulose was conducted as previously described with minor adaptations [57]. In brief, alcohol insoluble residues (AIR) were prepared from the different materials and used for all further analysis. Lignin was determined as acetyl bromide soluble lignin (ABSL) and crystalline cellulose content by Updegraff method both according to Foster et al. [58, 59]. Monosaccharide composition of the matrix polysaccharides in the AIR was determined after TFA hydrolysis by high-performance anion-exchange chromatography with pulsed amperometric detection (HPAEC-PAD) as described in Damm et al. [57]. The total acetate content was determined using an acetic acid kit (catalog #K-ACETRM, Megazyme, Wicklow, Ireland), as previously described [31].

### OrganoCat pretreatment

Plant biomass (100 g L^−1^) was suspended in a mixture of 0.1 M oxalic acid or FDCA (4 mL, as aqueous phase) and 2-MTHF (4 mL, as organic phase) in a stainless-steel high-pressure reactor, pressurized with argon (10 bar) [32]. The reactor was heated to 140 °C, 150 °C, or 160 °C, respectively, and maintained at the same temperature for 3 h. After cooling and depressurizing, the liquid phases were separated by decantation and the aqueous phase was filtered to isolate the cellulose-enriched pulp. The sugar concentration in the aqueous phase was determined using a reducing sugar assay by PAHBAH [60]. The solid residue was washed with distilled water until neutral pH and dried until constant weight. Lignin was obtained by evaporation of 2-MTHF. Reactions were conducted in triplicates.

### Saccharification test

Reactions were conducted in triplicates. Hydrolysis of raw material, Avicel, and pulp obtained from the OrganoCat pretreatment were carried out in an Eppendorf ThermoMixer Comfort using 1.5 mL Eppendorf vials. For each reaction, 20 mg material and 10 μL Accellerase 1500 (60 FPU mL^−1^ and 82 CBU mL^−1^, Genencor, The Netherlands) were dissolved in 1 mL of citrate buffer (pH = 4.5) and shaken at 50 °C for a specific time. Afterwards, samples were heated to 90 °C for 10 min to inactivate the enzymes. The glucose concentration was determined photometrically using a glucose (HK) assay kit obtained from Sigma-Aldrich. Glucose yields were calculated based on cellulose content as determined in the lignocellulose compositional analysis.

## ^1^H-^13^C HSQC-NMR measurements

The lignocellulosic material was washed with 70% ethanol, followed by a chloroform methanol mixture (1:1; v:v). The remaining AIR was destarched using α-amylase and Pullulanase [58]. The destarched lignocellulosic material and OrganoCat pretreated pulp was milled in a PM 100 CM ball mill using 50 ml Zirconium dioxide (ZrO_2_) vessel containing ZrO_2_ (10 × 10 mm) ball bearings, for a total time of 7 h (5 min grinding with 5 min pause interval at a speed of 600 rpm). The grinded material (25 mg) and the fractionated lignin material from the OrganoCat process were dissolved in 0.75 ml of DMSO-d_6_ (Sigma-Aldrich) containing 10 µl of EMIM (*O*Ac)-d_14_ [61] and stirred for 2 h (600 rpm) at 600 °C. The solutions were filled in an NMR tube and a 2D quantitative HSQC-NMR spectrum was recorded on a 600 MHz NMR spectrometer equipped with a cryoprobe. The following parameters were used to record the spectrum (pulse program: hsqcetgpsisp.2, NS: 200, inter-scan delay: 1 s, TD1: 2048 and TD2: 256 data points, temperature: 25 °C) as mentioned previously [61]. The NMR measurement and analysis were performed using the Bruker Topspin 3.6 software. The chemical shifts were referred to the solvent DMSO-d_6_ peak (δC 39.5 ppm, δH 2.49 ppm). Representative cross peaks were assigned according to the previous studies [61]. The anomeric signals of 3-*O*Ac- xylan, 2-*O*Ac-xylan, 2,3-*O*Ac-xylan, xylan, and α- and β-reducing end of xylan were summed up to 100%. The signals in the aliphatic region were integrated separately and the relative quantities of the 3-*O*Ac- xylan (H3-C3), 2-*O*Ac-xylan (H2-C2), and 2,3-*O*Ac-xylan (H3-C3) were quantified against the sum of their anomeric signals. The relative percentage of the monosaccharide composition was quantified by summing up the anomeric integrals of the monosaccharide signals to 100%. The polymer composition was quantified by summing up the integrals of the anomeric signals of cellulose and its reducing ends, anomeric signals of hemicellulose, and aromatic integrals of the lignin (S_2/6_/2, G_2_ and H_2/6_/2) to 100%. The lignin composition was quantified via the integration of ^13^C-^1^H correlations of the S_2/6_, G_2_ and H_2/6_ [62]. The integrals of S_2/6_/2, G_2_ and H_2/6_/2 were summed up to 100% and the relative percentage of S, G, and H lignin was quantified. The anomeric alpha (α) ^13^C–^1^H integrals of lignin linkages (β-aryl ether (A_α_/β-*O*-4), resinol (C_α_/(β-β), phenylcoumaran (B_α_/(β-5′), and dibenzodioxocin (D_α_/β-*O*-4′) linkages) were summed up to 100% and the relative percentage of the lignin linkages of A_α_, B_α_, C_α_, and D_α_ were quantified [31].

### Statistical analysis

The correlations between the different biomass and OrganoCat process factors were analyzed using Microsoft Excel 2013. The Pearson correlation coefficient and *p* value were used to evaluate the significance of each correlation. Principle component analysis was conducted using the free online tool ClustVis [63]. Prior to analysis, the data set was normalized by setting the highest value of each parameter to 1.0 and calculating the other value relative to the highest.

## Supplementary information

**Additional file 1.** Additional tables and figure.

## Data Availability

The datasets used and/or analyzed during the current study are available from the corresponding author on reasonable request.

## Abbreviations

ABSL: Acetyl bromide soluble lignin
AIR: Alcohol insoluble residue
Ara: Arabinose
CBU: Cellobiase units
DMSO: Dimethyl sulfoxide
EFB: Empty fruit bunch
EMIM: 1-Ethyl-3-methylimidazolium
FDCA: 2,5-furandicarboxylic acid
FPU: Filter paper units
G: Guaiacyl
GC: Gas chromatography
H: *p*-hydroxyphenyl
HPAEC: high-performance anion-exchange chromatography
HSQC: Heteronuclear single quantum coherence
MS: Mass spectrometry
2-MTHF: 2-methyltetrahydrofuran
NMR: Nuclear magnetic resonance
*O*Ac: *O*-acetyl
PAHBAH: 4-Hydroxybenzhydrazide
PAD: pulsed amperometric detection
PC: Principle component
S: Syringyl
TFA: Trifluoroacetic acid
Xyl: Xylose
