# Supplementary material for: Multiscale analysis of lignocellulose recalcitrance towards OrganoCat pretreatment and fractionation
Source: Biotechnol Biofuels. 2020 Sep 5;13:155. doi: 10.1186/s13068-020-01796-8 (PMC7487623; doi:10.1186/s13068-020-01796-8)

Additional Information:

Multiscale analysis of lignocellulose recalcitrance towards OrganoCat pretreatment and fractionation

Dennis Weidener[a,b,c‡], Murali Dama[c,d‡], Sabine K. Dietrich[a,c,e], Benedict Ohrem[a,c,], Markus Pauly[c,d], Walter Leitner[b,f], Pablo Domínguez de María[g], Philipp M. Grande[a,c], Holger Klose[a,c,e*]

[a] Institute of Bio- and Geosciences, IBG-2: Plant Sciences, Forschungszentrum Jülich, Leo- Brandt-Straße, 52425 Jülich, Germany

[b] Institute of Technical and Macromolecular Chemistry (ITMC), RWTH Aachen University, Worringer Weg 1, 52074 Aachen, Germany

[c] Bioeconomy Science Center (BioSC), c/o Forschungszentrum Jülich, 52425 Jülich, Germany

[d] Institute for Plant Cell Biology and Biotechnology, Heinrich Heine University, Universitätsstraße. 1, 40225 Düsseldorf, Germany

[e] Institute of Biology I, RWTH Aachen University, Worringerweg 1, 52074 Aachen, Germany,

[f] Max-Planck-Institut für Chemische Energiekonversion, Stiftstraße 34-36, 45470 Mülheim an der Ruhr, Germany

[g] Sustainable Momentum, SL. Av. Ansite 3, 4-6. 35006, Las Palmas de Gran Canaria, Spain

‡ These authors contributed equally to this work

*Corresponding author: Holger Klose, Tel.: +49 2461 61 3334; Fax: +49 2461 61 2492; E-mail address: h.klose@fz-juelich.de

Table S1: OrganoCat Pretreatment Mass fractions

	Catalyst	T [°C]	Pulp yield [wt%]	Error [wt%]	Pulp Hydrolysis [Glc wt%]	Error [wt%]	Lignin yield [wt%]	Error	Sugar yield	Error
								[wt%]	[wt%]	[wt%]
**Pineapple**	OA	140	51.66	1.59	51.40	1.49	7.54	2.04	13.23	0.98
	FDCA	160	38.90	1.83	53.24	2.22	16.48	2.59	14.67	0.74
	FDCA	150	43.50	0.44	45.39	3.94	13.78	0.71	23.69	1.94
	FDCA	140	46.14	2.59	43.84	6.45	6.92	0.34	18.62	4.21
**Beech**	OA	140	53.38	0.62	18.94	1.94	12.53	0.91	26.30	2.40
	FDCA	160	49.02	0.52	27.98	1.97	16.74	2.10	20.95	0.69
	FDCA	150	58.24	2.38	19.30	3.34	12.81	1.02	20.81	1.39
	FDCA	140	64.53	0.71	20.81	4.51	9.30	1.38	15.42	2.37
**Eucalyptus**	OA	140	53.83	0.69	20.30	1.20	0.38	1.50	15.29	1.09
	FDCA	160	50.84	3.87	32.56	3.35	0.17	0.68	10.90	1.29
	FDCA	150	55.58	1.30	19.52	3.03	0.13	0.53	13.10	1.70
	FDCA	140	62.18	3.62	8.29	0.83	0.15	0.59	8.46	0.89
**Maize**	OA	140	31.54	0.89	55.21	1.60	0.32	1.28	30.04	0.46
	FDCA	160	30.32	2.21	55.95	2.64	0.60	2.39	16.82	1.17
	FDCA	150	37.02	1.48	49.83	2.17	0.23	0.91	19.93	2.94
	FDCA	140	39.49	0.61	40.27	1.45	0.36	1.46	16.98	2.32
**Miscanthus**	OA	140	56.94	1.88	28.02	0.60	15.26	0.95	16.64	0.64
	FDCA	160	54.67	1.70	38.56	6.06	15.12	0.72	15.63	1.14
	FDCA	150	67.01	1.03	26.23	4.22	14.11	0.16	17.86	0.96
	FDCA	140	70.23	0.22	16.84	5.91	9.35	0.22	14.85	1.93
**Rapeseed**	OA	140	52.83	0.98	38.56	2.00	7.94	2.13	10.45	1.90
	FDCA	160	47.51	1.01	55.46	2.20	9.98	2.72	6.55	3.09
	FDCA	150	43.60	0.84	44.80	4.46	8.75	1.17	6.42	1.69
	FDCA	140	54.93	2.49	34.11	2.89	3.48	1.33	5.26	0.16
**Sida**	OA	140	54.03	2.19	37.12	1.15	9.96	0.91	16.39	2.42
	FDCA	160	53.06	1.90	44.61	3.84	13.21	3.35	10.00	0.89
	FDCA	150	59.91	1.25	31.04	2.19	7.70	1.06	8.28	1.18
	FDCA	140	67.25	1.00	20.17	1.55	4.99	2.19	7.11	1.19
**Silphium**	OA	140	53.91	0.77	21.24	4.17	11.79	2.40	5.85	0.71
	FDCA	160	39.06	1.66	37.37	0.36	14.99	2.32	5.87	0.75
	FDCA	150	43.13	0.42	24.81	0.31	12.97	1.38	5.40	0.59
	FDCA	140	53.96	2.21	20.24	0.09	6.33	0.71	4.79	0.91
**Sugarcane**	OA	140	47.69	0.92	53.55	3.18	16.55	2.75	17.33	2.57
	FDCA	160	45.26	1.94	60.75	4.08	18.74	2.27	11.93	3.21
	FDCA	150	50.92	0.70	55.96	3.94	10.74	0.98	13.56	0.59
	FDCA	140	56.28	1.25	48.15	1.13	11.10	0.57	13.93	1.14
**Szarvasi**	OA	140	35.06	2.22	56.73	2.14	15.35	1.15	24.80	1.66
	FDCA	160	32.53	1.17	65.04	1.31	19.43	1.17	16.02	2.98
	FDCA	150	39.03	0.29	54.98	4.08	16.09	0.87	21.87	1.70
	FDCA	140	42.84	0.87	41.90	7.03	11.70	2.10	18.73	3.26

Table S2: 2D-HSQC-NMR Data of lignocelluosic materials

Name/ (%)	Sida	Silphium	Beech wood	Szarvasi	Miscanthus	Maize cobs	Pineapple	Rapeseed	Sugarcane bagasse	Eucalyptus a)	EFB b)
**Polymer composition**											
cellulose	42.3±3.4	56.8±1.4	47.8±2.8	45.3±1.2	50.9±1.5	49.8±1.6	66.1±2.8	47.6±1.4	46.5±2.9	48.9	49.8±3.1
Hemicellulose	31.9±1.2	26.4±1.4	26.6±1.9	42.5±1.4	33.5±1.3	44.2±1.9	24.1±5.1	31.4±1.6	42.7±1.6	28.1	35.3±2.8
Lignin	25.8±2.3	16.9±0.1	25.6±1.5	12.2±0.9	15.6±0.7	6.0±1.0	9.8±2.7	21.1±2.8	10.8±1.4	22.9	14.9±0.5
**Monosaccharide composition**											
Glucose	57.0±3.1	68.3±1.3	64.2±2.9	51.6±1.4	60.3±1.6	53.0±1.9	73.4±4.9	60.2±0.7	52.1±2.5	48.9	58.2±3.8
Xylose	37.0±2.3	23.7±2.1	34.3±3.1	43.7±1.7	37.0±1.7	43.5±1.2	24.8±4.1	34.1±0.5	46.8±2.5	14.4	40.2±3.5
Arabinose	2.7±0.5	5.4±1.9	0.8±0.1	3.7±1.9	2.4±.12	3.0±1.5	0.5±0.1	3.1±0.4	0.4±0.1	1.8	0.4±0.1
4-OMe-GlcA	1.8±0.1	1.6±0.1	0.5±0.1	0.5±0.1	0.2±0.1	0.1±0.2	0.5±0.4	1.9±0.7	0.5±0.1	n.d	0.9±0.3
Mannose	1.4±0.4	1.1±0.5	0.3±0.1	0.6±0.1	0.1±0.2	0.4±0.1	0.8±0.7	0.7±0.4	0.3±0.1	0.6	0.4±0.1
**Lignin composition**											
Syringyl	51.4±0.7	48.9±2.0	68.1±1.3	36.2±1.6	32.7±0.5	32.5±0.8	8.4±6.2	41.0±2.4	43.1±2.6	77.0	54.1±0.7
Guaiacyl	48.3±0.5	41.6±0.5	31.9±1.3	57.8±1.5	57.8±1.5	48.8±2.5	83.1±9.0	57.5±2.1	54.6±2.5	23.0	42.5±0.7
p-Hydroxyphenyl	0.3±0.1	9.5±1.9	0.1±0.0	6.1±0.2	9.5±1.4	18.7±5.4	8.5±2.8	1.6±0.5	2.3±0.7	n.d	3.3±0.3
p-Coumarate(pCA)	n.d	n.d	n.d	3.6±0.8	15.1±1.4	20.3±5.3	n.d	n.d	21.8±8.0	n.d	n.d
Ferulate(FA)	n.d	n.d	n.d	5.7±0.7	8.1±0.6	21.3±1.4	n.d	n.d	18.3±7.0	n.d	n.d
**Lignin linkages composition**											
β-aryl ether (Aα)	86.2±2.1	83.7±2.9	81.7±1.1	71.9±3.3	68.3±0.5	65.8±3.3	94.5±2.4	87.8±1.4	73.8±5.7	69.0	90.5±1.1
Phenyl coumarin (Bα)	1.4±0.1	3.8±1.3	2.7±0.8	7.9±0.8	5.9±1.5	n.d	4.9±2.9	4.6±1.8	2.0±1.1	16.0	4.6±0.9
Resinol (Cα)	12.5±0.7	12.5±1.6	15.7±0.1	6.7±1.2	4.6±1.2	n.d	0.6±0.5	7.6±2.7	4.5±0.2	2.0	3.2±0.1
Dibenzodioxocin (Dα)	n.d	n.d	n.d	13.6±1.9	21.2±1.9	34.2±3.2	n.d	n.d	19.8±1.9	n.d	1.7±0.3
**O-Acetylation**											
2-OAc- Xylan	14.4±5.3	22.7±2.6	24.5±2.0	10.7±0.4	14.0±0.4	11.1±1.2	10.7±1.7	17.1±1.7	8.8±2.9	n.d	15.2±2.1
3-OAc-Xylan	49.5±2.8	41.6±5.0	41.7±4.8	19.8±0.9	26.8±1.9	19.8±0.1	28.3±4.9	44.1±0.4	23.5±2.0	n.d	39.5±0.6
2,3-OAc-Xylan	6.1±1.0	8.8±0.9	12.4±1.0	0.5±1.3	0.8±0.8	0.3±0.6	1.9±0.5	1.7±0.3	0.8±0.2	n.d	8.0±1.2
Total Ac-Xylan	70.1±3.8	73.1±6.9	78.6±2.6	30.9±0.8	41.6±2.6	31.2±0.8	40.8±3.6	62.9±2.1	33.2±1.8	55.0	62.8±2.3

[a] Eucalyptus composition reported from Rencoret et al. 2011 ; [b]EFB composition reported from Grande et al. 2019

Table S3: Wet-chemistry based characterisation of lignocelluosic materials

**Name/ (%)**	**Sida**	**Silphium**	**Beech wood**	**Szarvasi**	**Miscanthus**	**Maize cobs**	**Pineapple**	**Rapeseed**	**Sugarcane bagasse**	**Eucalyptus**	**EFB**
**Lignin (AcBr)**	20.7±3.4	19.8±1.4	30.7±2.8	23.8±1.2	30.6±1.5	30.8±1.6	25.1±2.8	23.2±1.4	32.4±2.9	48.9	49.8±3.1
**Cellulose (Seaman)**	51.4±1.2	20.4±1.4	41.6±1.9	42.9±1.4	43.4±1.3	40.8±1.9	51.9±5.1	50.4±1.6	47.4±1.6	28.1	35.3±2.8
**Acetic Acid**	25.8±2.3	16.9±0.1	25.6±1.5	12.2±0.9	15.6±0.7	6.0±1.0	9.8±2.7	21.1±2.8	10.8±1.4	22.9	14.9±0.5
**TFA-hydrolysable fraction**											
Total	*57.0±3.1*	*68.3±1.3*	*64.2±2.9*	*51.6±1.4*	*60.3±1.6*	*53.0±1.9*	*73.4±4.9*	*60.2±0.7*	*52.1±2.5*	*48.9*	*58.2±3.8*
Xylose	37.0±2.3	23.7±2.1	34.3±3.1	43.7±1.7	37.0±1.7	43.5±1.2	24.8±4.1	34.1±0.5	46.8±2.5	14.4	40.2±3.5
Arabinose	2.7±0.5	5.4±1.9	0.8±0.1	3.7±1.9	2.4±.12	3.0±1.5	0.5±0.1	3.1±0.4	0.4±0.1	1.8	0.4±0.1
Galactose	1.8±0.1	1.6±0.1	0.5±0.1	0.5±0.1	0.2±0.1	0.1±0.2	0.5±0.4	1.9±0.7	0.5±0.1	n.d	0.9±0.3
Mannose	1.4±0.4	1.1±0.5	0.3±0.1	0.6±0.1	0.1±0.2	0.4±0.1	0.8±0.7	0.7±0.4	0.3±0.1	0.6	0.4±0.1
Glucuronic Acid											

Table S4: Normalized data set pre pretreatment

Normalized data	Rapeseed	Sida	Silphium	EFB [a]	Miscanthus	Szarvasi	Maize	Sugarcane bagasse	Pineapple	Beech wood	Eucalyptus[b]
**Syringyl**	60.2	75.5	71.8	79.4	48.0	53.2	47.7	63.3	12.3	100.0	99.9
**Guaiacyl**	69.0	58.1	50.0	51.1	69.5	69.5	58.7	65.6	100.0	38.4	36.1
**Hydroxyphenyl**	8.8	1.6	51.1	17.6	50.8	32.6	100.0	12.4	45.3	0.5	10.7
**S/G**	31.5	46.9	51.9	56.2	25.0	27.6	29.4	34.9	4.5	94.2	100.0
**β-aryl ether**	92.9	91.2	88.6	95.8	72.3	76.1	69.6	78.0	100.0	86.5	n.d
**Phenyl coumarin**	58.3	17.7	48.1	58.2	74.7	100.0	0.0	25.2	62.1	34.2	n.d
**Resinol**	48.5	79.6	79.6	20.4	29.3	42.7	0.0	28.5	3.8	100.0	n.d
**Dibenzodioxocin**	0.0	0.0	0.0	5.0	62.0	39.8	100.0	57.8	0.0	0.0	n.d
**Xyl-*O*-Ac**	80.0	89.2	93.0	79.9	52.9	39.3	39.7	42.2	51.9	100.0	70.0
**Xyl/Ara**	8.6	10.5	3.4	77.3	11.9	9.1	11.1	100.0	36.2	33.0	n.d
**cellulose NMR**	72.0	64.0	86.0	75.4	77.1	68.6	75.4	70.4	100.0	72.4	74.0
**Hemicellulose NMR**	71.0	72.2	59.7	79.9	75.8	96.2	100.0	96.6	54.5	60.2	63.6
**Lignin NMR**	81.6	100.0	65.5	57.8	60.5	47.3	23.3	41.8	38.2	99.2	88.8
**ABSL**	71.5	63.9	61.1	56.1	94.4	73.6	95.1	100.0	77.4	94.4	80.3
**Seaman-Cellulose**	97.2	99.0	39.4	61.5	83.7	82.7	78.7	91.4	100.0	80.1	75.9
**total AcA**	81.1	57.9	50.7	100.0	42.6	31.7	51.2	46.9	67.0	70.8	50.4
**TFA-Xylose**	51.4	55.1	32.4	100.0	48.2	34.0	65.9	2.9	41.3	97.6	54.3

Table S5: Normalized data set post pretreatment

Normalized data	Rapeseed	Sida	Silphium	EFB [a]	Miscanthus	Szarvasi	Maize	Sugarcane bagasse	Pineapple	Beech wood	Eucalyptus[b]
**temperature dependency [slope]**	87.4	100	70.1	n.d	88.8	94.6	64.1	99.2	38.4	29.3	51.5
**Efficiency [extracted pulp]**	86.9	97.0	71.4	82.3	100	59.4	55.4	82.7	71.1	89.6	93.00
**degradability [Glc yield]**	75.9	61.1	51.1	100	52.8	89.0	76.6	83.2	72.9	38.3	44.5
**Extracted Lignin**	51.4	67.9	77.1	93.6	77.8	100	3.07	96.4	84.8	86.1	0.8
**p-hydroxyphenyl**	28.1	32.2	0	23.9	21.8	41.4	46.0	29.6	100	18.8	18.2
**guaiacyl**	73.3	70.9	91.0	71.5	98.1	100	85.0	86.8	51.8	54.0	48.5
**syringyl**	72.3	71.2	78.9	76.9	56.8	40.6	49.4	60.2	36.1	95.1	100
**ferulic acid**	47.3	72.5	90.1	0	68.3	89.7	100	54.1	60.8	52.9	37.6
**ß-O-4**	59.5	57.0	100	90.2	56.2	49.6	42.2	64.2	47.6	52.0	61.7
**ß-ß**	70.0	56.7	100	n.d.	32.2	34.0	15.5	17.2	22.0	71.0	73.3
**ß-5**	67.2	68.0	100	n.d.	85.5	92.4	47.8	50.8	46.4	61.9	64.1
**S/G**	47.8	48.7	42.1	50.3	28.1	19.7	28.1	33.6	33.8	85.3	100

Table S6: PCA Component loading “Biomass”

	PC1	PC2	PC3	PC4	PC5	PC6	PC7	PC8	PC9	PC10
**Syringyl**	0.3173	0.3031	-0.0232	0.0576	-0.1078	0.1074	-0.0414	0.1397	0.0748	-0.1064
**Guaiacyl**	-0.2653	-0.3611	0.2166	-0.0425	0.0556	-0.0673	0.0136	-0.1686	-0.0898	0.1174
**Hydroxyphenyl**	-0.2757	0.0198	-0.4955	-0.0703	0.1855	-0.1510	0.0895	0.0083	-0.0056	0.0019
**S/G**	0.3091	0.2544	-0.0962	0.0503	0.0633	0.2321	-0.2353	0.3719	-0.2089	-0.2891
**β-aryl ether**	0.2036	-0.4025	0.0653	0.1489	-0.0127	0.0613	0.1503	0.2118	-0.1756	-0.2220
**Phenyl coumarin**	-0.0308	-0.1994	0.0658	-0.2750	-0.5048	-0.0161	-0.6962	-0.1461	0.1310	-0.0690
**Resinol**	0.2903	0.1007	0.0951	-0.3291	0.0595	0.0284	0.1705	-0.5467	-0.3620	-0.1378
**Dibenzodioxocin**	-0.2803	0.3158	-0.1040	0.0727	0.0792	-0.0591	-0.0629	0.0921	0.2631	0.2491
**Xyl-O-Ac**	0.3696	-0.0743	-0.0916	-0.0565	0.1218	-0.0549	0.1921	-0.3155	0.2161	0.1488
**Xyl/Ara**	-0.0131	0.0263	0.1777	0.5555	-0.2236	0.5034	0.0216	-0.1904	-0.1380	0.4318
**cellulose NMR**	-0.0993	-0.3931	-0.3182	-0.0289	0.2480	0.3953	-0.1267	0.0355	-0.1073	-0.0670
**Hemicellulose NMR**	-0.2369	0.3050	0.0795	0.1990	-0.3581	-0.2636	0.1742	-0.1507	-0.1881	-0.3016
**Lignin NMR**	0.3497	0.0516	0.2202	-0.1868	0.1436	-0.0990	-0.0605	0.1187	0.3048	0.3882
**ABSL**	-0.1782	0.2813	0.1562	0.0677	0.4988	0.2839	-0.3326	-0.3854	0.1449	-0.2676
**Seaman-Cellulose**	-0.1097	-0.0780	0.6139	0.0824	0.3535	-0.2496	-0.0940	0.2491	-0.1383	-0.0846
**total AcA**	0.1956	-0.2436	-0.0683	0.5180	0.0173	-0.2193	-0.0186	-0.1996	0.4955	-0.3748
**TFA-Xylose**	0.2122	0.0104	-0.2597	0.3305	0.1811	-0.4649	-0.4325	-0.1258	-0.4508	0.2867

Table S7: PCA Component loading "Pretreatment"

	PC1	PC2	PC3	PC4	PC5	PC6	PC7	PC8	PC9	PC10
**temperature dependency [slope]**	0.1896	0.3481	0.1110	-0.4829	-0.2532	0.3105	0.1422	0.4356	-0.3172	0.2415
**Efficiency [extracted pulp]**	-0.2986	0.0468	0.2003	-0.6557	0.2546	0.2419	-0.2350	-0.1389	0.1605	-0.2931
**degradability [Glc yield]**	0.3136	0.0373	0.4633	0.1616	-0.4061	0.0478	0.3497	0.0888	0.1405	-0.4277
**Extracted Lignin**	0.1287	0.2252	0.4123	0.0907	0.7098	-0.3503	0.1186	0.3333	-0.0419	0.0322
**p-hydroxyphenyl**	0.2731	-0.4128	0.0976	0.1245	0.2621	0.5130	-0.0205	-0.0227	-0.1593	0.1048
**guaiacyl**	0.2817	0.4288	-0.1427	-0.1369	-0.1098	-0.3093	-0.0937	-0.2528	0.1830	0.3698
**syringyl**	-0.4555	0.0698	0.0133	-0.0155	-0.1619	-0.2486	0.0867	0.2039	0.0143	-0.3643
**ferulic acid**	0.2294	0.0851	-0.6463	0.0273	0.1335	0.0077	-0.1255	0.4359	-0.1666	-0.4165
**ß-O-4**	-0.1751	0.3861	0.2674	0.4246	-0.1275	0.1869	-0.6377	0.0190	-0.3145	-0.0273
**ß-ß**	-0.3495	0.2251	-0.1301	0.2691	0.0696	0.4140	0.2125	0.3266	0.5574	0.2739
**ß-5**	-0.0358	0.4787	-0.1630	0.1205	0.2306	0.2694	0.4437	-0.5153	-0.2516	-0.2477
**S/G**	-0.4380	-0.1711	-0.0086	0.0060	-0.0577	-0.1429	0.3272	0.0581	-0.5407	0.2841

Figure S8: Correlation of S/G ratio to β-*O*-4 linkages

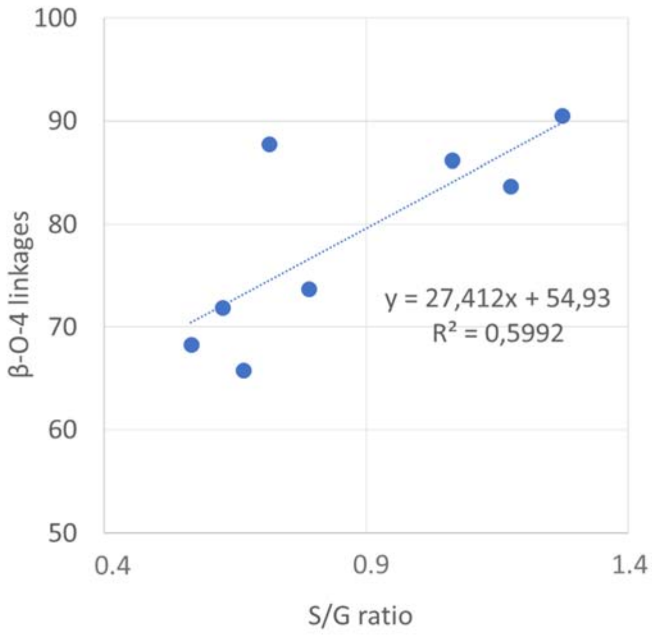

Supplement: Supplementary file 1 — Additional file 1. Additional tables and figure. [file 13068_2020_1796_MOESM1_ESM.pdf]
